# Machine learning-assisted system using digital facial images to predict the clinical activity score in thyroid-associated orbitopathy

**DOI:** 10.1038/s41598-022-25887-8

**Published:** 2022-12-21

**Authors:** Jae Hoon Moon, Kyubo Shin, Gyeong Min Lee, Jaemin Park, Min Joung Lee, Hokyung Choung, Namju Kim

**Affiliations:** 1 Department of Internal Medicine, Seoul National University Bundang Hospital and Seoul National University College of Medicine, Seongnam, Republic of Korea; 2 Center for Artificial Intelligence in Healthcare, Seoul National University Bundang Hospital, Seongnam, Republic of Korea; 3 THYROSCOPE INC., Ulsan, Republic of Korea; 4 Department of Ophthalmology, Seoul National University Bundang Hospital and Seoul National University College of Medicine, 82, Gumi-ro 173 Beon-gil, Bundang-gu, Seongnam-si, Gyeonggi-do 13620 Republic of Korea; 5 Department of Ophthalmology, Hallym University Sacred Heart Hospital and Hallym University College of Medicine, Chuncheon, Republic of Korea; 6 Department of Ophthalmology, Seoul Metropolitan Government Seoul National University Boramae Medical Center and Seoul National University College of Medicine, Seoul, Republic of Korea

**Keywords:** Endocrinology, Translational research, Machine learning, Eye manifestations

## Abstract

Although the clinical activity score (CAS) is a validated scoring system for identifying disease activity of thyroid-associated orbitopathy (TAO), it may produce differing results depending on the evaluator, and an experienced ophthalmologist is required for accurate evaluation. In this study, we developed a machine learning (ML)-assisted system to mimic an expert’s CAS assessment using digital facial images and evaluated its accuracy for predicting the CAS and diagnosing active TAO (CAS ≥ 3). An ML-assisted system was designed to assess five CAS components related to inflammatory signs (redness of the eyelids, redness of the conjunctiva, swelling of the eyelids, inflammation of the caruncle and/or plica, and conjunctival edema) in patients’ facial images and to predict the CAS by considering two components of subjective symptoms (spontaneous retrobulbar pain and pain on gaze). To train and test the system, 3,060 cropped images from 1020 digital facial images of TAO patients were used. The reference CAS for each image was scored by three ophthalmologists, each with > 15 years of clinical experience. We repeated the experiments for 30 randomly split training and test sets at a ratio of 8:2. The sensitivity and specificity of the ML-assisted system for diagnosing active TAO were 72.7% and 83.2% in the test set constructed from the entire dataset. For the test set constructed from the dataset with consistent results for the three ophthalmologists, the sensitivity and specificity for diagnosing active TAO were 88.1% and 86.9%. In the test sets from the entire dataset and from the dataset with consistent results, 40.0% and 49.9% of the predicted CAS values were the same as the reference CAS, respectively. The system predicted the CAS within 1 point of the reference CAS in 84.6% and 89.0% of cases when tested using the entire dataset and in the dataset with consistent results, respectively. An ML-assisted system estimated the clinical activity of TAO and detect inflammatory active TAO with reasonable accuracy. The accuracy could be improved further by obtaining more data. This ML-assisted system can help evaluate the disease activity consistently as well as accurately and enable the early diagnosis and timely treatment of active TAO.

## Introduction

Thyroid-associated orbitopathy (TAO) is the most common extrathyroidal symptom of autoimmune thyroid diseases, including Graves’ disease and autoimmune thyroiditis. Clinically obvious TAO occurs in 30–50% of patients with Graves’ disease and less frequently (0.1%–0.3%) in patients with chronic autoimmune thyroiditis. Subclinical orbital lesions can be observed by magnetic resonance imaging in most patients with Graves’ disease^1–4^. The autoimmune inflammation of orbital tissues induced by thyroid autoantibodies results in periorbital edema, restrictive strabismus, and proptosis^5,6^. The treatment of TAO depends on the clinical activity and severity, as assessed by standardized criteria, and comprises predominantly immunosuppressive therapy including intravenous glucocorticoids in patients with inflammatory active TAO of moderate to severe severity^7^. However, such immunosuppressive therapy is not effective in patients with fibrous changes in the orbits caused by chronic inflammation, and more serious sequelae occur if timely treatment is not provided during the active phase of TAO^1,8^. Therefore, early identification of patients at risk of active TAO and prompt implementation of appropriate treatment are important for improving the prognosis of TAO^1,7,8^.

The best validated scoring system for assessing TAO activity is the clinical activity score (CAS)^7^. The CAS, which was proposed in 1989, aimed to distinguish the active inflammatory phase of TAO, when immunosuppressive therapy is expected to be effective^9^. The modified CAS is used most widely. This scoring system comprises seven items with binary answers (yes/no), and TAO is defined as active if the score is ≥ 3^10^. Each item of the CAS relates to the inflammatory symptoms and signs of TAO, including five signs of orbital redness and swelling and two symptoms of orbital pain^10^. Although the CAS is intuitive and easy to apply, it may produce differing results depending on the evaluator, and an experienced ophthalmologist is required for accurate evaluation.

Recently, along with the development of machine learning (ML) methodologies in the field of artificial intelligence (AI), several AI solutions have been developed to help diagnose various diseases by reading medical images such as X-ray images or computed tomography, and some have already been commercialized^11^. These diagnostic aid AI solutions learns and mimics expert image reading, demonstrating expert-level medical image reading capabilities. If this can be applied to TAO diagnosis, it is possible to develop AI solution to mimic an expert’s CAS assessment using a patient’s digital facial image. Moreover, this AI solution can be combined with various smart devices, and it can provide patients a more accessible method for evaluating the disease activity consistently as well as accurately and enable the early diagnosis and timely treatment of active TAO.

In this study, we developed a machine learning (ML)-assisted system to mimic an expert’s CAS assessment and predict the CAS using digital facial images obtained from 1,020 patients with TAO. We evaluated the accuracy for predicting the CAS and diagnosing active TAO using this ML-assisted system to explore the potential as a useful screening tool for active TAO in the patients with autoimmune thyroid disease.

## Results

### Participant characteristics

The baseline characteristics of the 1,020 patients included in this study are shown in Table 1. The mean age was 45.2 ± 15.4 years, and 57.2% (411 of 719) were women. Anti-thyrotropin (TSH) receptor antibody was present (> 1.0 U/mL) in 88.7% of the patients. The mean CAS was 2.0 ± 1.3, and the most common inflammatory symptom or sign was swelling of the eyelids. Active TAO (CAS ≥ 3) was observed in 272 patients (29.6%), and highly active TAO (CAS ≥ 5) in 34 (3.7%). Because orbital pain was not recorded for 102 patients, the CAS was calculated for 918 patients.

**Table 1:** Baseline characteristics.

Age (year)	45.2 ± 15.4
Sex (male/female)	301/719
BMI (kg/m^2^)	23.1 ± 3.6
**Thyroid function test**	
Free T4 (ng/dL)	1.63 ± 1.27
TSH (mIU/L)	0.07 (0.72)
Anti-TSH receptor antibody	4.6 (27.7)
CAS (total score)*	2.0 ± 1.3
Spontaneous retrobulbar pain (yes/no)*	109 / 809
Pain on attempt up- and down gaze (yes/no)*	78 / 840
Redness of eyelids (yes/no)	342 / 678
Redness of conjunctiva (yes/no)	428 / 592
Swelling of eyelids (yes/no)	849 / 171
Inflammation of the caruncle and/or plica (yes/no)	123 / 897
Conjunctival edema (yes/no)	107 / 913

Data are expressed as mean ± SD or median (interquartile range).

*BMI* body mass index, *T4* thyroxine, *TSH* thyrotropin, *CAS* clinical activity score.

*918 patients were included in analysis due to lack of record about orbital pain.

### Diagnostic performance of the ML-assisted system for identifying active TAO and each inflammatory sign

The performance of our ML-assisted system for diagnosing active TAO and each inflammatory sign included in the CAS when the test set was constructed from the entire dataset are shown in Table 2. The system had sensitivity of 72.7% and specificity of 83.2% for diagnosing active TAO. In the test set constructed from the dataset with consistent results of the three ophthalmologists (Table 3), the sensitivity and specificity for diagnosing active TAO were 88.1% and 86.9%, respectively. For each inflammatory sign assessed by the ML-assisted system using the test set from entire dataset, the highest area under the curve (AUC) was for redness of the conjunctiva (80.9%) and the lowest was for inflammation of the caruncle and/or plica (75.0%). In the test set constructed from the dataset with consistent results, the highest AUC was for inflammation of the caruncle and/or plica (97.9%) and the lowest was for swelling of eyelids (88.4%).

**Table 2:** Diagnostic accuracy of a ML-assisted system for active TAO and each inflammatory sign of CAS in the test set constructed from entire dataset.

	Sensitivity (95% CI), %	Specificity (95% CI), %	AUC (95% CI), %
Active TAO (CAS ≥ 3)	72.7 (70.6, 74.8)	83.2 (81.7, 84.8)	
Redness of eyelids	63.7 (61.7, 65.7)	75.3 (73.9, 76.6)	77.1 (76.0, 78.3)
Redness of conjunctiva	71.8 (70.4, 73.1)	73.4 (71.6, 75.3)	80.9 (80.0, 81.8)
Swelling of eyelids	84.8 (83.8, 85.8)	50.6 (47.2, 53.9)	77.1 (75.6, 78.6)
Inflammation of the caruncle and/or plica	42.6 (38.6, 46.5)	90.6 (89.6, 91.5)	75.0 (73.2, 76.8)
Conjunctival edema	52.9 (49.1, 56.7)	88.0 (87.0, 89.0)	79.1 (77.2, 81.0)

*ML* machine learning, *CI* confidence interval, *AUC* area under the curve, *TAO* thyroid associated orbitopathy, *CAS* clinical activity score.

**Table 3:** Diagnostic accuracy of a ML-assisted system for active TAO and each inflammatory sign of CAS in the test set constructed from the dataset with consistent results.

	Sensitivity (95% CI), %	Specificity (95% CI), %	AUC (95% CI), %
Active TAO (CAS ≥ 3)	88.1 (86.7, 89.6)	86.9 (86.0, 87.8)	
Redness of eyelids	79.7 (77.1, 82.3)	85.1 (84.3, 85.9)	91.2 (90.4, 92.0)
Redness of conjunctiva	79.5 (77.6, 81.4)	84.2 (83.2, 85.2)	89.6 (88.8, 90.4)
Swelling of eyelids	84.2 (83.5, 85.0)	76.6 (74.3, 78.8)	88.4 (87.8, 89.0)
Inflammation of the caruncle and/or plica	97.5 (94.7, 100.0)	88.3 (87.7, 89.0)	97.9 (97.5, 98.4)
Conjunctival edema	98.9 (96.7, 100.0)	86.2 (85.2, 87.2)	95.8 (95.3, 96.3)

*ML* machine learning, *CI* confidence interval, *AUC* area under the curve, *TAO* thyroid associated orbitopathy, *CAS* clinical activity score.

### Prediction of the CAS

The distributions of the predicted CAS generated by our ML-assisted system and reference CAS evaluated by the ophthalmologists are shown in Tables 4 and 5. In the test set constructed from the entire dataset, 40.0% (95% confidence interval (CI) 38.7–41.2) of the predicted CAS values were the same as the reference CAS values (Table 4). In the dataset constructed with the consistent results, 49.9% (95% CI 48.7–51.2) of the predicted CAS values were the same as the reference CAS values (Table 5). The ML-assisted system predicted the CAS to within 1 point of the reference CAS in 84.6% (95% CI 83.8–85.4) and 89.0% (95% CI 88.0–90.0) of patients when tested using the entire dataset and the dataset with consistent results, respectively.

**Table 4:** Prediction of the CAS by the ML-assisted system using the test set constructed from the entire dataset.

	Predicted CAS								
Reference CAS		0	1	2	3	4	5	6	7
	0	214	156	101	16	4	3	0	0
	1	253	733	589	144	41	2	0	0
	2	74	349	713	324	86	20	1	0
	3	21	84	219	308	227	54	1	0
	4	7	49	63	178	161	86	19	2
	5	0	1	12	29	42	44	25	11
	6	0	0	0	0	2	1	17	0
	7	0	0	0	0	0	1	3	7

All of 5497 test results from 30 repeated experiments were presented. The predicted CAS was generated using the ML-assisted system, and the reference CAS was evaluated by three ophthalmologists using the patients’ facial images.

*CAS* clinical activity score, *ML* machine learning.

**Table 5:** Prediction of the CAS by the ML-assisted system using the test set constructed from the dataset with consistent results.

	Predicted CAS								
Reference CAS		0	1	2	3	4	5	6	7
	0	231	183	79	0	0	0	0	0
	1	234	509	325	76	4	0	0	0
	2	28	88	507	126	114	10	0	0
	3	0	5	43	114	118	3	0	0
	4	0	0	10	16	42	17	0	0
	5	0	0	0	0	0	68	22	0
	6	0	0	0	0	0	0	27	0
	7	0	0	0	0	0	0	0	0

All of 3,000 test results from 30 repeated experiments were presented. The predicted CAS was generated using the ML-assisted system, and the reference CAS was evaluated by three ophthalmologists using the patients’ facial images.

*CAS* clinical activity score, *ML* machine learning.

## Discussion

In this study, we developed a ML-assisted system to assess the CAS in patients with TAO and detect active TAO using patients’ digital facial images. Our ML-assisted system showed reasonable performance as a screening tool for “referable” TAO for early diagnosis. The incidence and severity of TAO in patients with Graves’ disease have decreased^3,12–14^ because of the reduction in smoking, better control of thyroid dysfunction, and early diagnosis of TAO^7^. Interaction between endocrinologists and ophthalmologists is important for the early diagnosis of TAO. Endocrinologists should consider the possibility of TAO and refer patients to an ophthalmologist because mild TAO can progress to more severe disease requiring expert advice and guidance, and timely treatment can improve the prognosis and quality of life in patients with TAO^2,15^. The treatment of TAO is based on its activity, severity, and disease duration because anti-inflammatory or immunosuppressive treatment is less effective for inactive disease or disease of ≥ 18 months duration, and the risk of treatment outweighs the benefit in patients with mild disease^7,16,17^. Therefore, moderate to severe TAO in the active phase is a major target of treatment, and all patients with active disease are recommended to be evaluated by an ophthalmologist for proper management.

Diabetic retinopathy (DR) is another condition for which it is important to refer patients to an ophthalmologist for timely treatment. ML-assisted systems for detecting referrable DR using digital fundus photographs have recently been developed. In 2018, the US Food and Drug Administration (FDA) permitted marketing of the first AI-based medical device, called IDx-DR, for detecting referrable DR in adult patients with diabetes. Considering that the performance thresholds for IDx-DR were defined as 85.0% for sensitivity and 82.5% for specificity, the performance of our ML-assisted system for detecting active TAO when tested with the entire dataset (72.7% sensitivity and 83.2% specificity) needs further improvement. However, the performance using the test set constructed from the dataset with consistent results of the three ophthalmologists (88.1% sensitivity and 86.9% specificity) may be adequate for US FDA approval as a screening system. Moreover, in the patients with highly active TAO (CAS ≥ 5), our system detected 93.3% and 100% of active TAO in the experiments using the entire dataset (Table 1) and the dataset with consistent results (Table 2), respectively. According to the natural history of TAO described by Rundle^2,18,19^, anti-inflammatory therapy is indicated in the middle of the dynamic phase of TAO, when inflammatory activity peaks. The results presented here suggest that our ML-assisted system may successfully detect the patients who are in optimal period of treatment of their TAO.

Our ML-assisted system showed differences in performance for each inflammatory sign. Although the final goal of our ML-assisted system is to be useful for the diagnosis of active TAO based on the sum score of the seven CAS items, the ability to detect each inflammatory sign must be improved for future applications. In the test of the ML-assisted system using the test set constructed from the entire dataset, the sensitivity for each inflammatory sign correlated highly with the prevalence of each sign in the dataset (Supplementary Table S1). This result suggests that the performance of our system was affected by the amount of positive data, such as the number of facial images with specific inflammatory signs. Therefore, further data acquisition and data preprocessing, such as data augmentation, should improve the performance of this system. On the other hand, it is difficult to read all five signs of orbital inflammation accurately, even for experienced ophthalmologists. As shown in Supplementary Table S2, the consistency rate for the positivity of each sign as assessed by the three ophthalmologists was < 50% for all signs. The CAS scoring system can be used to correct the reading error for each sign by calculating the final score by combining the assessments of the five signs and two pain symptoms. Implementing this correction process for the CAS scoring system, as shown here using our ML-assisted system, improved the accuracy of detecting active TAO (CAS ≥ 3), and this accuracy was better than that for detecting each inflammatory sign.

Our ML-assisted system showed better performance when tested using the dataset with consistent results for the three ophthalmologists. The higher performance in the test set with consistent results suggests that our system learned the typical characteristics of each sign well and successfully mimicked the ophthalmologists’ reading. For example, inflammation of the caruncle and/or plica is generally rare, and its prevalence was low in our dataset (12.1%). Because ophthalmologists know about its low prevalence, this sign on a facial image will be read as positive only when the image shows typical changes in the caruncle and/or plica. Moreover, if all three ophthalmologists give a positive result for a facial image, that image must contain characteristic findings. The sensitivity and AUC of our system for inflammation of the caruncle and/or plica were the lowest among the five signs when tested using the entire dataset. However, when tested using the dataset with consistent results, the sensitivity and AUC increased. These results suggest that our ML-assisted system successfully learned these typical findings of inflammation of the caruncle and/or plica.

This study has strengths that the proposed system can detect the early stage of TAO based on CAS before serious changes in appearance occur. Recently, two studies to diagnose TAO from digital facial images using AI models were published^20,21^. Karlin et al.^20^ reported a deep learning-based classifier to identify the presence of TAO, not the active phase of TAO which can be derived from CAS, based on facial images where the classifier is the ensemble of ResNet 18 networks. Using the major feature of TAO that the disease may result in facial disfigurement, they could achieve accuracy of 89.2% (sensitivity 93.4%; specificity 86.9%). Huang et al.^21^ reported a diagnostic system to identify each individual sign of TAO using deep learning-based sematic segmentation network (to detect ocular dyskinesia) and classification network (to detect other signs). Their model showed diagnostic performances of 0.60–0.94 of AUC according to the signs of TAO. Our work has three differences compared two aforementioned studies: (1) labels on all facial images were re-evaluated by three experienced ophthalmologists, not according to medical records, (2) we developed two consensus models (aggregating model and voting model) to mimic the discussion process of these three specialists, rather than just adopting a conventional network, and (3) we aimed to develop a system to estimate CAS, the best validated scoring system for assessing TAO activity, and detect inflammatory active TAO. Therefore, the system we proposed is based on more solid data and performs a more useful task in the real clinical practice.

This study has important clinical implications. Including our ML-assisted system in a medical mobile app that can be installed on the patient’s smartphone will allow detection of active TAO using “selfie” images taken by the patient. The mobile app should then be able to advise a patient with active TAO to visit an ophthalmologist. This solution would enable TAO monitoring in daily life. In addition, our ML-assisted system can be applied to telemedicine because doctor–patient consultations are generally conducted through video calls. The ML-assisted system embedded in the telemedicine device may be used to evaluate CAS using the patient’s facial image, and physicians such as general practitioners or endocrinologists can use this information to refer patients with active TAO to an ophthalmologist at an early stage. These applications of our ML-assisted system, including self-monitoring of TAO activity and diagnostic assistance for referable TAO, will provide clinical value for improving the prognosis of TAO through early diagnosis and timely treatment.

This study has some limitations that should be considered when interpreting the results. First, the amount of data was insufficient for an ideal AI solution. Given the nature of AI solutions, performance should improve with an increase in data. Therefore, further investigations and development involving a larger amount of data are warranted to improve this ML-assisted system. Second, all submodels in our ML-assisted system adopted a support vector machine (SVM). Although we cropped the regions of interest (ROIs) from facial images using the image preprocessing module, SVMs ignore a key property of images—that nearby pixels are more strongly correlated than are more distant pixels because SVMs treat the image as the input to a fully connected network^22^. To overcome this limitation, a convolutional neural network or vision transformer can be applied in future investigations.

In conclusion, this study showed that it is possible to assess CAS with an AI solution using digital facial images. As more data are obtained, this system’s performance may improve to the level of an experienced ophthalmologist. This technology should enable patients to self-monitor their TAO activity and help clinicians identify patients needing referral. The use of this technology for screening and the timely treatment of active disease could be expected to help improve the prognosis and quality of life of patients with TAO.

## Methods

### Study design and dataset

This single-center, retrospective study used digital facial image datasets and medical records from 1020 patients with TAO who visited the outpatient clinic of the ophthalmology department at Seoul National University Bundang Hospital (SNUBH) from January 2010 to October 2021. Patients aged 18 years or older with TAO whose CAS was evaluated by an ophthalmologist (NK) were eligible for inclusion. Their digital facial images were obtained at a distance of one meter using a digital single-lens reflex camera (Canon EOS 80D, 2976 × 1984 resolution, Tokyo, Japan). The participant was instructed to immobilize his or her head with the eyes in the primary position, and the head position was examined to confirm the absence of obvious tilt or chin-up or chin-down position.

This study was approved by the SNUBH Institutional Review Board (IRB# B-2109-709-101), and the requirement for informed consent was waived by SNUBH Institutional Review Board given that the study design was based on a retrospective review of medical records and pre-obtained facial images. All experiments were performed in accordance with relevant guidelines and regulations. All images and clinical data were anonymized before being transferred outside the hospital, and the process of data transfer was approved by the SNUBH Data Review Committee (DRB-2021-02-01).

### CAS scoring

Three oculoplastic specialists, each with > 15 years of clinical experience at tertiary referral hospitals, interpreted the facial images and reevaluated the CAS according to the criteria suggested by the European Group on Graves’ Orbitopathy (EUGOGO)^10,23^. The CAS comprises seven items, each answered “yes” or “no”, that ask about spontaneous retrobulbar pain, pain on attempted up and down gaze, redness of the eyelids, redness of the conjunctiva, swelling of the eyelids, inflammation of the caruncle and/or plica, and conjunctival edema. The final score is the sum of all items (maximum score of 7), and active TAO was defined as a CAS ≥ 3. Each ophthalmologist independently interpreted the patients’ facial images and rated the five items relating to orbital swelling and redness by referring to a standard photographic color atlas^24^. For each item for all patients, the result was recorded as is if the three ophthalmologists gave a consistent score. If the scores from three ophthalmologists were not consistent, they held a discussion until reaching consensus. For the two items relating to orbital pain, information in the medical records was used. The score for each CAS item and the final score after this scoring process were used as the reference values for training and testing the ML-assisted system. Among the 1020 patients included in this study, 102 had no record of orbital pain. Therefore, the facial images for 1020 patients were used in training and testing for detecting each inflammatory sign, and data for 918 patients were used in the test process for predicting CAS and diagnosing active TAO.

### Development of the ML-assisted system

**Figure 1:**
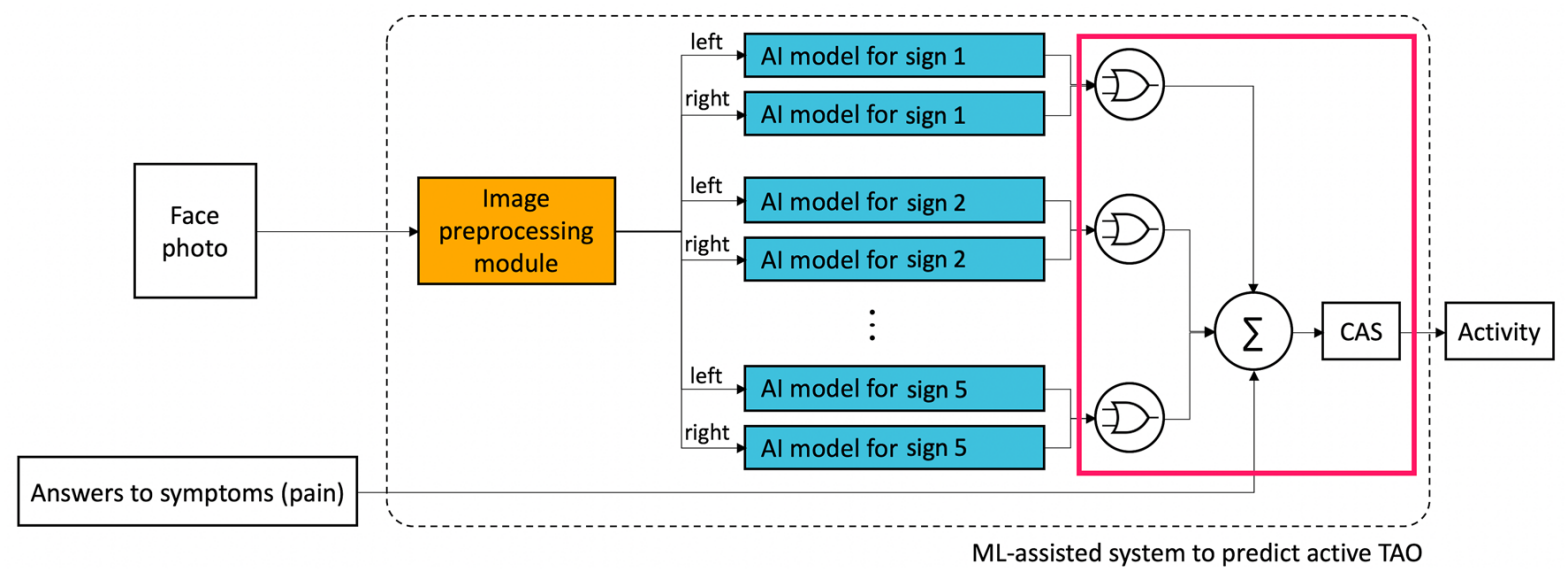
Structural diagram of the ML-assisted system to predict active TAO. *AI* artificial intelligence, *CAS* clinical activity score, *ML* machine learning, *TAO* thyroid-associated orbitopathy.

Our proposed ML-assisted system is designed to simulate the process used by an ophthalmologist to obtain the CAS and diagnose active TAO. The process comprises three steps: (1) determine the presence or absence of each of the five inflammatory signs of orbital swelling and redness, including redness of the eyelids, redness of the conjunctiva, swelling of the eyelids, inflammation of the caruncle and/or plica, and conjunctival edema) in each eye; (2) determine whether the patient has the sign based on the result of each eye (whether at least one eye has the sign); and (3) calculate the CAS and identify the clinical activity of TAO. Figure 1 shows the structure of our ML-assisted system which comprises components corresponding to the processes mentioned above, and an image preprocessing module. The inputs of this system include the patient’s digital facial images and answers to two symptoms about orbital pain, and output is the activity of the patient’s TAO.

The image preprocessing module (depicted in the orange box in Fig. 1) crops the ROI from the inserted facial image so that only the region where the inflammatory sign appears can be viewed intensively when judging each sign. This module identifies the face in the image and then detects key facial landmarks that can be used as criteria for cropping. Finally, for each eye, three cropped images are generated for the following areas: the orbital region (to observe the redness/swelling of eyelids), the lateral part of the conjunctiva (to observe the redness/edema of conjunctiva), and the medial part of the conjunctiva and caruncle (to observe the redness of the conjunctiva and inflammation of the caruncle and/or plica).

**Figure 2:**
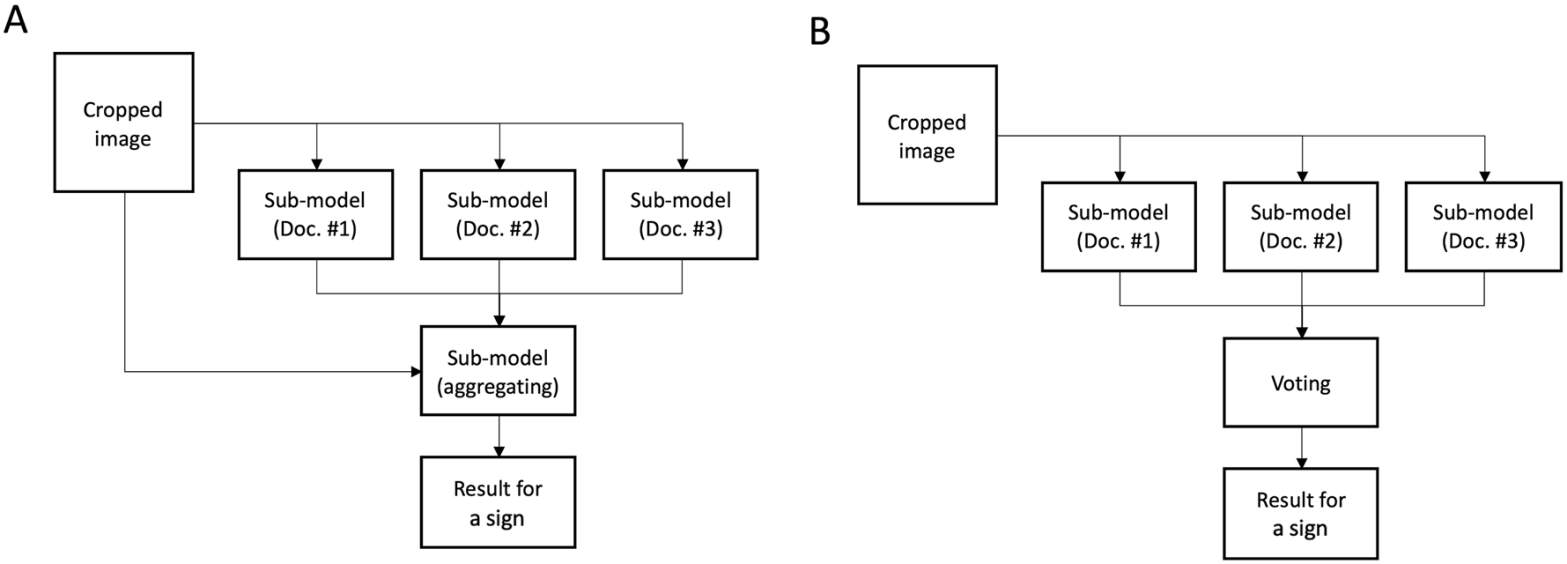
Structural diagram showing the aggregating model (**A**) and voting model (**B**).

To predict the five signs of orbital redness and swelling, five AI models (depicted in the blue boxes in Fig. 1) were created for which each model predicted the presence of an inflammatory sign in each eye using a cropped image. Each AI model predicting each sign adopted one of two consensus models (aggregating model and voting model, Fig. 2) by using the individual diagnosis of each ophthalmologist and their agreed final diagnosis.

The aggregating model was designed to mimic the discussion of the three ophthalmologists (Fig. 2A). First, three submodels predicted the presence of a sign using an image, just as the three ophthalmologists individually diagnosed this. Each submodel was trained using a cropped image as an input and each ophthalmologist’s diagnosis for each inflammatory sign as an output. Finally, the aggregating model produced a consensus result using the input image and the results from the former submodels. This aggregating model was trained using a cropped image and each ophthalmologist’s diagnosis for a sign as inputs and the agreed diagnosis from three ophthalmologists as an output. The aggregating model was adopted in the AI models to predict redness of the eyelids and inflammation of the caruncle and/or plica according to experiment results.

The voting model was inspired by the observation that most of the final diagnosis results were the same as those of the majority vote based on the individual diagnoses of the ophthalmologists (Fig. 2B). The voting model decided on the presence of a sign using the majority of results from the three submodels that simulated each ophthalmologist’s diagnosis. The voting model was adopted in the AI models to predict swelling of the eyelids and redness/edema of the conjunctiva according to the experiment’s results.

For both aggregating model and voting model, linear kernel SVM integrated with linear kernel PCA was adopted for all submodels. To address the overfitting problem, the hyperparameters, regularization parameter C for SVM and the number of components for PCA, were tuned via fivefold cross validation.

The last part of the ML-assisted system (depicted within the box outlined in red in Fig. 1) determines whether the patient had each inflammatory sign based on the predicted result for each eye. The system then calculates the CAS using the information about the two pain-related symptoms to predict the TAO activity. A patient is considered to show the sign if the inflammatory sign was found in at least one eye. Finally, if three or more symptoms and signs are determined to be present (i.e., predicted CAS ≥ 3), the system classifies the patient as being in the active phase of TAO.

All experiments were conducted on the computer running Ubuntu 19.04.6 LTS equipped with AMD Ryzen Thredripper 3990 × 64-core processor CPU, 256 GM of RAM and two NVIDIA GeForce RTX 3090 GPUs.

### Anthropometric and biochemical measurements

We measured the patients’ height and weight while wearing light clothing and without shoes to the nearest 0.1 cm and 0.1 kg, respectively. Body mass index was calculated as the weight divided by the square of the height as is expressed in kg/m^2^. Serum concentrations of free thyroxine (T4) and TSH were measured using immunoradiometric assays (free T4: DiaSorin S.p.A.; TSH: CIS Bio International). The reference ranges for free T4 and TSH were 0.89–1.79 ng/dL and 0.3–4.0 mIU/L, respectively. Patients were examined for the presence of anti-TSH receptor antibody using a radioimmunoassay (Cis Bio International), and the cutoff for positivity was > 1.0 U/mL.

### Statistical analysis

Values with a normal distribution are expressed as mean ± SD, and values with a nonnormal distribution are expressed as median (interquartile range). The sensitivity and specificity of the ML-assisted system for detecting each CAS item and active TAO were computed based on the reference diagnoses of the three ophthalmologists. Receiver operating characteristic curve analysis was performed to calculate the AUC of the ML-assisted system for detecting each CAS item. The 95% CI for each statistical value was also calculated. The diagnostic values were analyzed using the test set from the entire dataset and from the dataset in which all three ophthalmologists gave consistent results. Each analysis was performed in 30 experiments. The sensitivity, specificity, and AUC of the ML-assisted system are presented as mean value and 95% CI of the results of 30 experiments. The experiments to test the entire dataset were conducted for randomly split training and test sets in the ratio of 8:2. The experiments to test the dataset in which all three ophthalmologists gave consistent results were performed on 100 patients randomly selected as the test set of the 132 patients with consistent results. The remaining 920 patients (32 with consistent results and 888 with inconsistent results) were included in the training set.

## Supplementary Information

Supplementary Information.

## Data Availability

The raw data including medical records and facial images are not available upon request due to ethical and legal restrictions imposed by the SNUBH Institutional Review Board. The original data are derived from the institutions’ electronic health records and contain patients’ protected health information. Deidentified data are available from the SNUBH for researchers who meet the criteria for access to confidential data and have a data usage agreement with the hospital.

## Abbreviations

TAO: Thyroid associated orbitopathy
CAS: Clinical activity score
ML: Machine learning
AI: Artificial intelligence
TSH: Thyrotropin
T4: Thyroxine
AUC: Area under the curve
CI: Confidence interval
DR: Diabetic retinopathy
FDA: Food and drug administration
SVM: Support vector machine
PCA: Principal component analysis
ROI: Region of interest
SNUBH: Seoul National University Bundang Hospital
EUGOGO: European group on Graves' orbitopathy
