# Supplementary material for: Machine learning-assisted system using digital facial images to predict the clinical activity score in thyroid-associated orbitopathy
Source: Sci Rep. 2022 Dec 21;12:22085. doi: 10.1038/s41598-022-25887-8 (PMC9772205; doi:10.1038/s41598-022-25887-8)
Supplement: Supplementary file 1 — Supplementary Information. [file 41598_2022_25887_MOESM1_ESM.docx]

| **Supplementary Table 1.** Prevalence of each inflammatory sign of CAS in the dataset | |
| --- | --- |
|  | Prevalence (%) |
| Redness of eyelids | 33.5 |
| Redness of conjunctiva | 42.0 |
| Swelling of eyelids | 83.2 |
| Inflammation of the caruncle and/or plica | 12.1 |
| Conjunctival edema | 10.5 |
| CAS, clinical activity score | |

| **Supplementary Table 2.** Prevalence of each inflammatory sign of CAS in the dataset | | | |
| --- | --- | --- | --- |
|  | Reference result | Results from three ophthalmologists | |
|  |  | consistent (%) | not consistent (%) |
| Redness of eyelids | positive | 31.0 | 69.1 |
|  | negative | 68.7 | 31.3 |
| Redness of conjunctiva | positive | 43.2 | 56.8 |
|  | negative | 72.3 | 27.7 |
| Swelling of eyelids | positive | 47.9 | 52.1 |
|  | negative | 29.2 | 70.8 |
| Inflammation of the caruncle and/or plica | positive | 14.6 | 85.4 |
|  | negative | 83.5 | 16.5 |
| Conjunctival edema | positive | 6,5 | 93.5 |
|  | negative | 92.0 | 8.0 |
| CAS, clinical activity score | | | |
